# Pathogenicity of a novel bovine adenovirus type 3 with a natural deletion partial fiber gene in BALB/c mice

**DOI:** 10.3389/fvets.2023.1138159

**Published:** 2023-03-23

**Authors:** Qian Li, Shufan He, Yuantong Zou, Hua Yue, Cheng Tang, Jie Liu

**Affiliations:** ^1^College of Animal and Veterinary Sciences, Southwest Minzu University, Chengdu, Sichuan, China; ^2^Key Laboratory of Qinghai-Tibetan Plateau Animal Genetic Resource Reservation and Utilization, Southwest Minzu University, Chengdu, Sichuan, China

**Keywords:** bovine adenovirus type 3, pathogenicity, deleted strain, fiber gene, BALB/c mice

## Abstract

**Objective:**

A novel Bovine adenovirus type 3 (BAdV-3) with a natural deletion partial fiber gene was isolated in 2020 and named BO/YB24/17/CH. The objective of this study was to understand the pathogenicity of this virus.

**Methods:**

Thiry-two 3-week-old BALB/c mice were divided into three experimental groups and a control group. Mice in the experimental groups were intranasally inoculated with virus, and mice in the control group were inoculated with MDBK cell supernatant. Mice were weighed and clinically examined daily for appearance of any clinical signs. Three infected mice and one control mouse were euthanized at 1, 3, 5, 7, 9, 11, 13, and 15 days after inoculation. Tissue samples were collected for histopathological examination, immunohistochemical staining, and detection of the replication dynamics of virus.

**Results:**

All infected mice developed mild clinical signs such as lethargy, weight loss, loss of appetite, and a rough hair coat, and gross lesions were observed as pulmonary punctate hemorrhage, lobular atrophy and splenomegaly. Histopathological examination revealed thickening of alveolar septa and mildly dilated splenic nodules and blurred red-white medullary demarcation in the spleen. Immunohistochemical results further confirmed that the production of the above lesions was due to viral infection. Importantly, unlike previously reported BAdV-3 detection only in the lungs and trachea, this isolate could be detected in multiple organs such as the heart, liver, spleen, kidney, and even blood by virus isolation and titration and real-time PCR methods.

**Clinical significance:**

This study provides further insight into the pathogenicity of the fiber region deletion strain BO/YB24/17/CH in BALB/c mice, which provides a reference for the prevention and control of BAdV-3 as well as the development of vaccines.

## 1. Introduction

Bovine adenovirus type 3 (BAdV-3) is an important pathogen causing bovine respiratory disease and is commonly involved in bovine respiratory disease complex (BRDC) in feedlot cattle (1–3). BRDC is a type of serious bovine respiratory disease caused by mixed infection with multiple pathogens, and causes huge economic losses to the cattle industry worldwide every year (4, 5).

BAdV-3 is an unenveloped double stranded DNA virus belonging to the genus *Mastadenovirus* of the family *Adenoviridae*. BAdV-3 can cause a variety of clinical signs, such as fever, pneumonia, bronchitis and other respiratory symptoms. The intensity of the signs is related to the age of infection, with adult cattle usually showing no or mild upper respiratory signs, while calves show significant clinical signs (6, 7). The pathological changes in BAdV-3 infected cattle were mainly confined to the lungs, characterized by inflammatory cell infiltration, necrotizing bronchitis, type II alveolar cell proliferation, and alveolar septa thickening (8–11). Since Darbyshire et al. isolated BAdV-3 from the conjunctiva of healthy dairy cows in the UK in 1965, it has been detected in many countries, geographically including Asia, Europe, North America, South America and Africa (4, 7, 12–15). Serological detection showed positive rates of BAdV-3 antibodies in cattle in Belgium, Finland, Iran, Poland and Turkey ranging from 46.7 to 92.3% (5, 16–19), indicating that BAdV-3 has been widely prevalent in the world.

**Figure 1:**
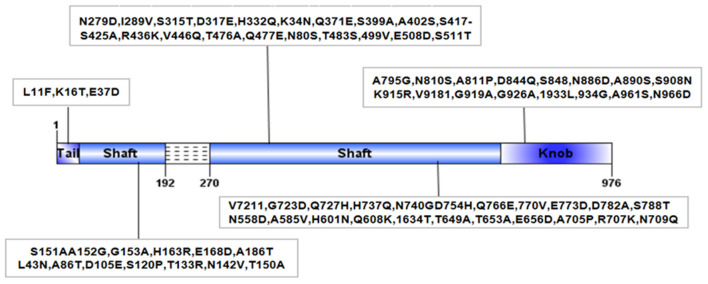
Structural diagram of fiber protein of BO/YB24/17/CH strain. Dashed lines indicated amino acid deletion at position 192~270 of BO/YB24/17/CH in shaft region. The black box indicated the unique amino acid mutation of BO/YB24/17/CH compared to all available complete 9 BAdV-3 fiber sequences.

Recently, our laboratory conducted pathogenic tests on 11 introduced cattle farms with BRDC symptoms in Sichuan China, and the results showed that the positive rate of BAdV-3 was as high as 78.7%. Unexpectedly, a BAdV-3 strain, named BO/YB24/17/CH, was isolated from nasal swabs. Analysis of the intact fiber of the isolate revealed 79 amino acid deletions in the shaft domain and 74 unique amino acid mutations compared with the available complete sequences for fiber genes in the GenBank database (Figure 1). Furthermore, it was confirmed that BAdV-3 with the natural deletion partial fiber gene has a wide geographical distribution in China (14). Considering that fiber is the major structural component of the adenovirus capsid that interacts with the cellular receptor and determines host cell tropism, and that deletions and mutations in this region have previously been reported to affect the pathogenicity of adenovirus (20, 21), further studies of this isolate are warranted. Currently, natural infections with BAdV-3 in calves are ubiquitous, thus making it difficult to find suitable calves for experimental infection. Therefore, to understand the pathogenicity of this novel BAdV-3 infection, 24 specific-pathogen-free (SPF) BALB/c mice were inoculated intranasally with BO/YB24/17/CH. This study provides further insight into the pathogenicity of the fiber region deletion strain in BALB/c mice, which provides a reference for the prevention and control of BAdV-3 as well as the development of vaccines.

## 2. Material and methods

### 2.1. Virus and laboratory animals

BAdV-3 strain BO/YB24/17/CH was propagated and titrated in MDBK cell monolayers grown in DMEM medium with 10% fetal bovine serum, and the virus titer was 10^6.32^ 50 % tissue culture infective doses per ml (TCID_50_/mL). Thirty-two specific-pathogen-free (SPF) BALB/c female mice (3 weeks old) were purchased from Chengdu Dashuo Laboratory Animal Co., Ltd.

### 2.2. Experimental infection of BALB/c mice with strain BO/YB24/17/CH

Thiry-two 3-week-old SPF BALB/c mice were divided into three experimental groups (*n* = 8 per group) and a control group (*n* = 8). After anesthesia by ether, mice in the experimental group were intranasally inoculated with 200 μL of virus (virus titer: 10^6.32^ TCID_50_/mL), and mice in the control group were intranasally inoculated with the same volume of uninfected MDBK cell supernatant. Three infected mice (one from each experimental group) and one control mouse were euthanized by ether inhalation followed by cervical dislocation at 1, 3, 5, 7, 9, 11, 13, and 15 days after inoculation. Each tissue of the mice was divided into three parts: one for virus titration, one for histopathological examination and immunohistochemical staining, and one for real-time PCR. Blood was collected from mice at each time point for further detection of the virus by virus titration and real-time PCR.

### 2.3. Clinical observations, virus isolation, and titration

Mice were weighed and clinically examined daily for appearance of any clinical signs, such as feed intake, mental status, nasal discharge, etc. After dissection under aseptic conditions, heart, liver, spleen, lung, kidney, and trachea samples were weighed, and 100 mg of each tissue sample was homogenized in 1.5 ml tubes containing 0.9 mL DMEM and cryopreserved at−80°C for virus isolation and titration. Freeze-thaw was repeated twice, and the processed homogenate was centrifuged at 5,000 rpm for 10 min to harvest the supernatant. Virus was isolated by using MDBK cells as follows: serial 10-fold dilutions of tissue homogenates were made in DMEM supplemented with 2% fetal bovine serum. MDBK cells cultured in 96-well cell culture plates were seeded with each dilution in 4 wells and incubated in a 5% CO_2_ incubator at 37°C for 5–7 days. TCID_50_/ml of tissue homogenate was calculated by the method of Reed and Muench.

### 2.4. Detection of viral DNA by real-time PCR

For constructing the absolute quantitation real-time PCR for BAdV-3, standard curves were generated based on the pMD19-T plasmid (Takara, China) containing a 120-bp sequence of the BAdV-3 hexon. The concentration of the positive standards was measured with a micro nucleic acid protein concentration analyzer, and diluted with a 10-fold gradient. The copy number of the standards was converted according to the formula: copy number = plasmid concentration × 10^−9^ × 6.02 × 10^23^/660 × total plasmid length), and then the standard curve was constructed using GraphPad Prism 8.

Total DNA was extracted from 100 mg of each tissue homogenate using a Viral DNA Kit (Tiangen, China) according to the manufacturer's instructions. BAdV-3 was detected by a specific real-time PCR assay previously established in our laboratory (14). This assay can only specifically amplify the hexon gene of BAdV-3. BAdV-3-120 (F:5′-ACTCCTACAAAACCCGCTTCC-3′, R: 5′- CGCTGTAAGGCTTGAAACT-3′)was used to amplify a 120-nucleotide (nt) region of the hexon gene (nucleotides 18,014~18, 133 bp in the BAdV-3 prototype strain WBR-1 genome sequence, GenBank accession number AC_000002.1). The amplification was conducted in a 20 μl reaction volume containing 0.05 μM forward primer, 0.05 μM reverse primer, 2 μl of DNA, 10 μl of TB Green Premix Ex Taq II (2 × Premix) (TaKaRa Bio Inc.), and an appropriate volume of double-distilled water. The virus copy number of each tissue sample was converted by the standard curve.

### 2.5. Histopathology and immunohistochemistry

The 4% paraformaldehyde fixed tissues were embedded in paraffin and subjected to serial sections (5 um). After deparaffinizing in xylene, the sections were rehydrated in graded alcohols, and stained with hematoxylin and eosin (H&E) or used for immunohistochemistry (IHC). The primary antibody used for IHC staining was a rabbit anti-BAdV-3 polyclonal antibody (produced in our laboratory) at a dilution ratio of 1:800, and was applied overnight at 4°C. The secondary antibody was horseradish peroxidase (HRP)-labeled anti-rabbit IgG (Proteintech, China) at a dilution of 1 to 1,000 and used for 30 min at 37° C. The images of H&E and IHC were analyzed with a light microscope (Nikon, Tokyo, Japan).

### 2.6. Statistical analysis

Values are means ± SD unless otherwise indicated. Statistical differences between groups were determined using one-way ANOVA. *P* < 0.05 was considered significant.

## 3. Results

### 3.1. Clinical signs and gross lesions in BALB/c mice infected with BO/YB24/17/CH

**Figure 2:**
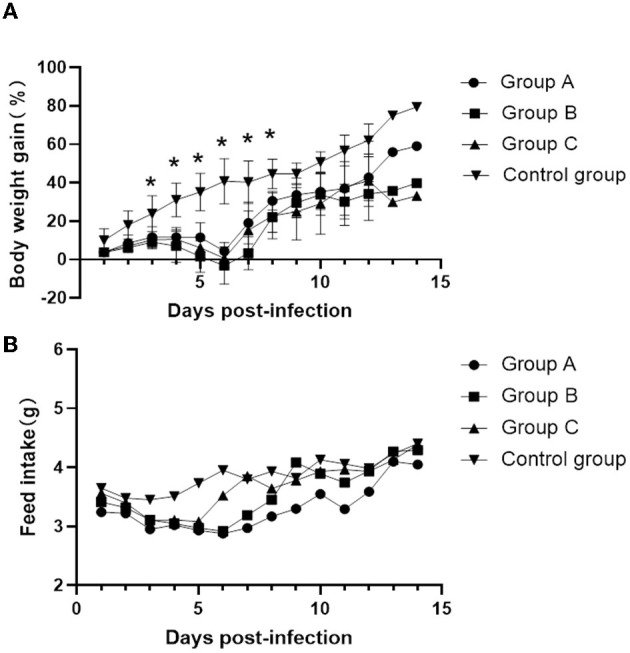
Changes in body weight gain and food intake of mice at different times after inoculation. **(A)** Daily weight gain. **(B)** Feed intake. Values are mean ± SD. *P < 0.05 (by one-way ANOVA).

**Figure 3:**
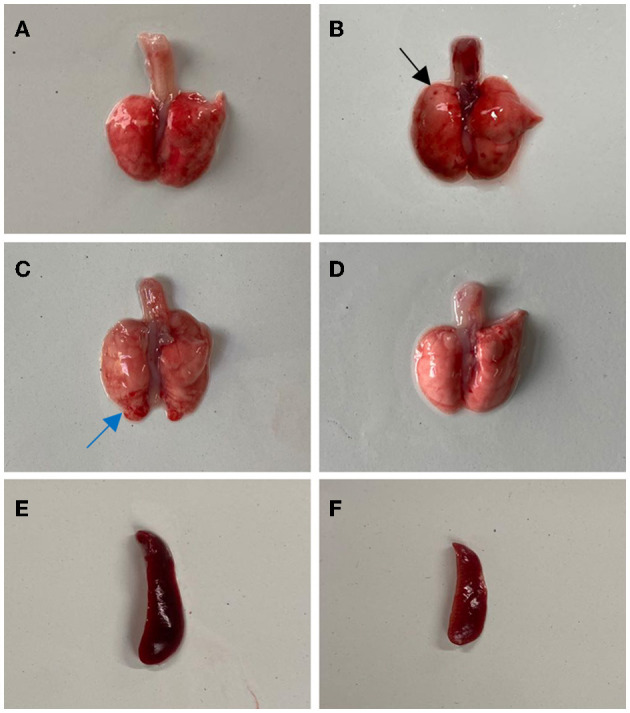
Gross lesions in BALB/c mice after infection with BO/YB24/17/CH strain. **(A)** Lung congestion (the 3th day after infection). **(B)** Lung hyperemia with sporadic petechial, indicated with black arrow (the 11th day after infection). **(C)** Pulmonary hemorrhage and lobular atrophy, indicated with blue arrow (the 15th day after infection). **(D)** Lung of the control group. **(E)** Spleen swelling (the 5th day after infection). **(F)** The spleen of the control group.

From 3 to 7 days after inoculation, infected mice developed mild clinical symptoms such as lethargy, weight loss, loss of appetite, and a rough hair coat, and no typical respiratory symptoms were observed throughout the observation period. Compared with the control group, mice in the infected group showed slower body weight gain on day 3 post-infection (PI) and gradually recovered after reaching the minimum value on day 6 (Figure 2A). Correspondingly, feed intake in the infected group was reduced from days 2 to 6 and gradually recovered after day 7 (Figure 2B). Macroscopic observation showed congestion in the lungs of the infected mice on day 3 PI (Figure 3A), scattered pulmonary hemorrhagic spots on day 11 PI (Figure 3B), and massive hemorrhage and lobular atrophy in the lungs on day 15 PI (Figure 3C). Notably, swelling of the spleens and darkening of the spleens were observed in infected mice from day 5 PI (Figure 3E). According to the statistics, a total of 17/24 mice in the infected group showed pathological changes in the lungs and 8/24 mice showed pathological changes in the spleens throughout the infection period (Table 1). No macroscopic lesions were present in the control mice at necropsy (Figures 3D, F).

**Table 1:** Gross pathological changes in the infected mice.

**Dissection time (days)**	**Number of dissected mice**	**Pathological changes of lung**	**Pathological changes of spleen**
1	3	1/3	0/3
3	3	2/3	0/3
5	3	2/3	1/3
7	3	2/3	2/3
9	3	3/3	1/3
11	3	2/3	2/3
13	3	3/3	1/3
15	3	2/3	1/3
Total	24	17/24	7/24

### 3.2. Histopathological examination and immunohistochemistry staining

**Figure 4:**
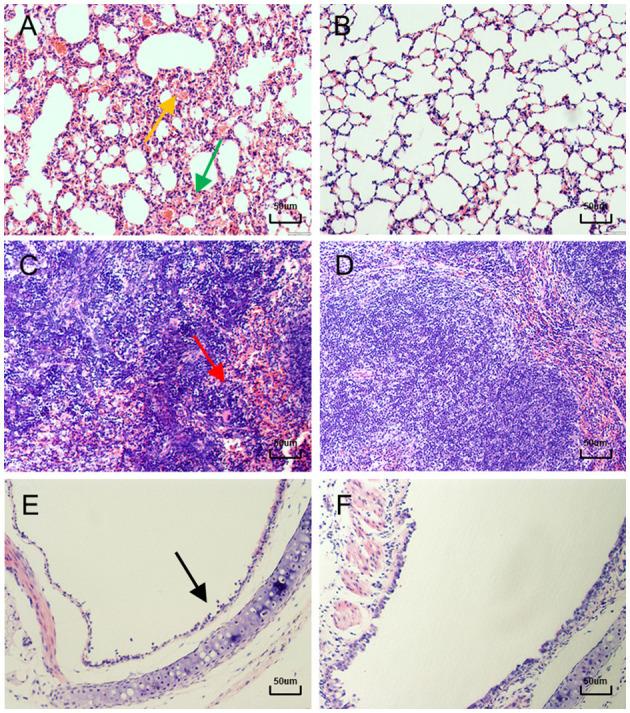
Lung, Spleen and Trachea sections of mice after hematoxylin and eosin (H&E) staining. **(A)** 11 days after infection, the lungs of mice showed (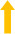) marked congestion and hemorrhage, and (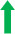) alveolar septal thickening (**400×**). **(B)** Normal control lung sections from mock-inoculated mice did not show observable histological changes (**400×**). **(C)** 5 days after infection, the spleens of mice showed (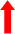) blurred red-white medullary demarcation (**400×**). **(D)** Normal control spleen sections from mock-inoculated mice did not show observable histological changes (**400×**). **(E)** 13 days after infection, the tracheas of mice showed (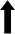) detached epithelial cells (**400×**). **(F)** Normal control trachea sections from mock-inoculated mice showing no observable histological changes (**400×**).

By histopathological examination, compared with the control mice, mice in the infected group exhibited marked congestion and hemorrhage in the lungs, which induced thickening of alveolar septa from day 3 to day 15 PI (Figures 4A, B). These lesions caused a decreasing of the gas exchange area. Mildly dilated splenic nodules and blurred red-white medullary demarcation were observed in the spleens of infected mice from day 5 to day 15 PI, whereas no abnormalities were observed in the spleens of the control mice (Figures 4C, D). From day 13 PI, detached epithelial cells were visible in the tracheas of the infected mice, while the control mice showed a normal histology structure (Figures 4E, F). Moreover, no significant lesions were observed in other organs of infected mice such as heart, liver and kidney.

**Figure 5:**
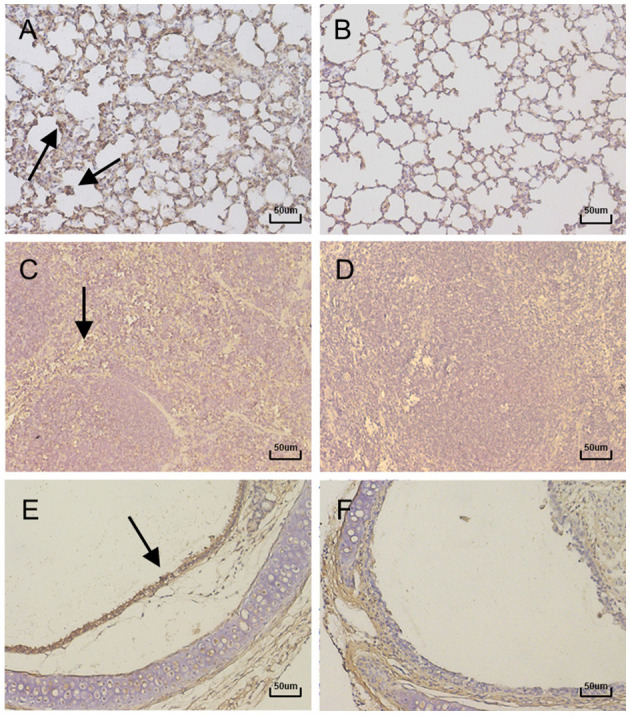
Immunohistochemistry of lung, Spleen and Trachea sections of mice (400×). Representative histological sections from lung tissue of mice at 3 days PI. **(A)** lung tissue of control. **(B)** Spleen tissue at 5 days PI. **(C)** Spleen tissue of control. **(D)** Trachea tissue at 13 days PI. **(E)** Trachea tissue of control. **(F)** were stained with immunohistochemical (IHC) using antibody specific for BAdV-3. Positive signals were indicated with black arrows.

In order to further confirm that the production of the above lesions was due to virus infection, immunohistochemistry was performed on the lungs, spleens, and tracheas using a specific polyclonal anti-BAdV-3 antibody. The results showed positive signals in the lungs, spleens and tracheas of infected mice (Figures 5A, C, E), but no positive signals were detected in the control group (Figures 5B, D, F). Specific signals were detectable in the lungs of infected mice throughout the experimental period, with a peak value occurring at day 3 PI. From the tropism of BAdV-3, the virus mainly infected bronchioles and alveolar epithelial cells (Figure 5A). Positive signals for BAdV-3 could be detected in the marginal areas of the red and white pulp of the spleens and the tracheal mucosa of infected mice on days 5 and 13 after inoculation, respectively (Figures 5C, E).

### 3.3. Virus replication kinetics in BALB/c mice infected with BO/YB24/17/CH

**Figure 6:**
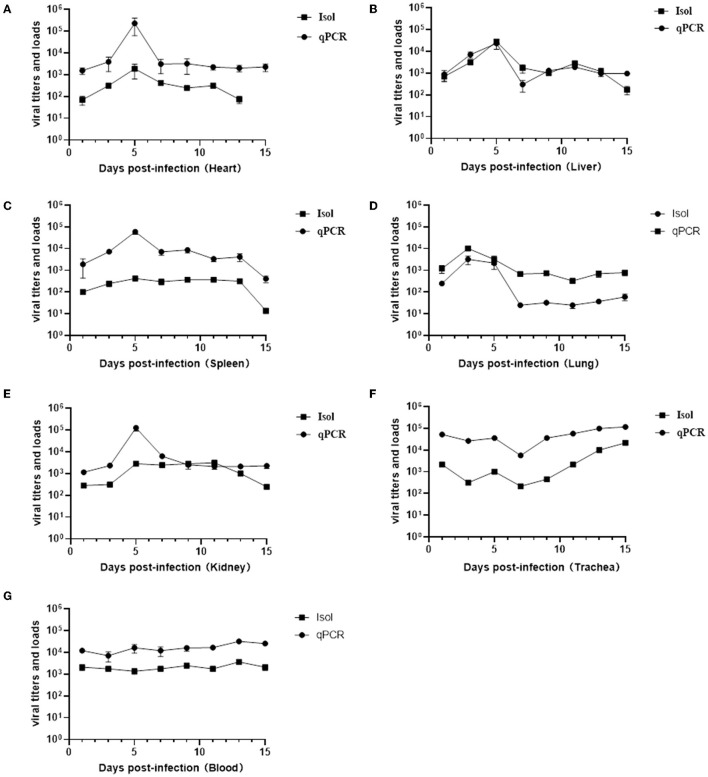
Results of viral titers (TCID_50_) and viral loads in various organs and blood at different infection time. Virus titration and viral load changes in the hearts **(A)**, livers **(B)**, spleens **(C)**, lungs **(D)**, kidneys **(E)**, trachea **(F)**, and blood **(G)**.

To determine whether the tissue tropism of the virus was altered, the replication kinetics of the virus in various tissues and organs were examined by virus isolation and titration and absolute quantitation real-time PCR. The results of viral titers and viral loads were summarized in Supplementary Table S1 and line graphs were constructed based on the data (Figure 6). Unlike previous reports where the virus could only be detected in the lungs and trachea (22, 23), in this study it was detected in multiple organs and even in the blood throughout the infection period. With the exception of the trachea and blood, the replication of the virus in all organs showed a certain regularity, reaching the highest viral titers and viral loads on a given day and then gradually decreasing. The viral titers and viral loads in the lungs reached the highest on the third day after infection, at 10^4.67^ TCID_50_/ml and 1.45 × 10^5^ copies/g, respectively, consistent with the strongest positive signal detected by immunohistochemistry on day 3 (Figure 6D). The heart, liver, spleen, and kidney showed the highest viral titers and viral loads on the day 5 (Figures 6A–C, E). Viral titers and viral loads maintained a stable and slightly increasing trend in the blood, indicating that the virus can cause viremia (Figure 6G). No viral DNA was detected in the control mice.

## 4. Discussion

Currently, the use of cattle as experimental animals is the most direct research method to study bovine respiratory diseases. However, due to the high cost, long reproductive cycle and high natural infection rate of BAdV-3 in cattle, it is very difficult to obtain appropriate experimental cattle for experimental infection. Therefore, SPF animals may be an effective model for the study of BAdV-3. Mitta et al. intranasally inoculated BAdV-3 into cotton rats and found that the virus was able to undergo limited replication in the lungs and trachea, consistent with the symptoms of BAdV-3 infection in cattle (24). Shi Hongfei et al. studied the pathogenicity of Chinese BAdV-3 strain HLJ0955 by intranasal inoculation into BALB/c mice and guinea pigs and found that the infection model would serve as a useful system for monitoring the infection process and pathogenesis (22, 23). These suggest that SPF experimental animals are feasible as an animal model to study BAdV-3. Therefore, the present paper reports the pathogenicity of a novel bovine adenovirus type 3 with a natural deletion partial fiber gene in BALB/c mice. Since the HLJ0955 strain was not isolated and preserved by our laboratory, there was no way to carry out infection experiments with the wild type strain as a control and comparisons could only be made with previously reported pathogenicity data, which is a drawback of this study.

Although the BO/YB24/17/CH isolate was not lethal to mice in infection experiments, it induced mild clinical signs such as lethargy, weight loss, loss of appetite, and a rough hair coat. Gross lesions observed in the lungs of mice showed the same hemorrhagic spots as those in BALB/c mice and albino guinea pigs infected with the HLJ0955 strain, but unique signs of lobular atrophy were also found in the lungs on day 15 of infection (22, 23). Notably, swelling of the spleen and darkening of the spleen were observed in infected mice from day 5 PI. These signs have not been reported in experimental animals previously infected with BAdV-3. Since spleen is the major immune organ, splenic lesions suggest that infection with this strain may have affected the immune response in mice. Recent reports have shown that the adenovirus-encoded E1A gene assembles into a multimeric complex with host proteins to mediate RAS signaling, thereby affecting the activation of interferon-stimulated genes (ISGs) and suppressing the host innate immune response (25). It has also been reported that infection of rhesus monkeys with host-range mutant human adenovirus type 5 suppressed their innate immune response and induced systemic CD4+ T cell activation (26), suggesting that mutations in adenovirus have the potential to affect the immune response of the host. Histopathological examination revealed marked hemorrhage, thickening of alveolar septa, inflammatory cell infiltration, and alveolar epithelial necrosis in the lung, and damaged cilia and epithelial cell detachment in the trachea, which were similar to previous pathogenicity experiments in calves, cotton rats and albino guinea pigs (10, 13, 22, 24). Immunohistochemistry experiments were also conducted to further confirm that the production of the above lesions was due to virus infection. However, the results were not very satisfactory, as the antigen-specific signal was not very strong. Possible reasons are on the one hand since there are no commercial anti-BAdV-3 antibodies, the antibody used for IHC staining was produced by our laboratory and it may not be very effective. On the other hand it is also possible that the amount of virus in the tissues is not sufficient to be detected. There is no doubt that the method needs further optimization.

Subsequently, the replication kinetics of the virus in various tissues and organs was detected. The results of virus titration and qPCR showed that the virus could be detected as early as 24 h after infection, and maintained high viral loads from 1 to 15 days, with a much longer duration of infection than previously reported for HLJ0955 strain (22, 23). In addition, the virus could infect and replicate in various organs, such as heart, liver, spleen, lung, kidney, trachea and even blood, which was different from the previous report that the virus replicated only in the lung and trachea (22–24), indicating that the virus tissue tropism has been altered and can cause viremia. This may be related to the deletion of the fiber shaft domain, which has been previously shown to play a decisive role in viral tissue tropism and immune evasion by the length of the shaft domain and the variation of the knob domain (20, 21, 27, 28). Undoubtedly, further work is needed to evaluate how deletion of the fiber shaft domain alters the tissue tropism of this isolate and how it affects the host immune response.

In summary, the above results demonstrated that the novel strain BO/YB24/17/CH with a natural deletion fiber gene was able to replicate in BALB/c mice and could cause clinical signs and gross and histological lesions in infected mice. Moreover, the isolate has a broader tissue tropism and is capable of causing viremia.

## Data availability statement

The datasets presented in this study can be found in online repositories. The names of the repository/repositories and accession number(s) can be found in the article/Supplementary material.

## Ethics statement

The animal study was reviewed and approved by the Animal Ethics Committee of the College of Animal and Veterinary Sciences, Southwest Minzu University.

## Author contributions

SH, CT, and JL developed the concept and designed the studies. SH and QL carried out the major part of study, analyzed data, interpreted results of experiments, prepared figures, drafted manuscript, and edited. JL revised manuscript and approved final version of manuscript. YZ and HY participated some parts of the study. All authors contributed to the article and approved the submitted version.
