# Supplementary material for: Pathogenicity of a novel bovine adenovirus type 3 with a natural deletion partial fiber gene in BALB/c mice
Source: Front Vet Sci. 2023 Mar 23;10:1138159. doi: 10.3389/fvets.2023.1138159 (PMC10076824; doi:10.3389/fvets.2023.1138159)
Supplement: Supplementary file 1 [file Table_1.DOC]

| **DPI^b^** | **Tests** | **Heart** | **Liver** | **Spleen** | **Lung** | **Kidney** | **Trachea** | **Blood** |
| --- | --- | --- | --- | --- | --- | --- | --- | --- |
| **1** | **TCID_50_**  **qPCR** | **3(10,10^2^,10^2^)** | **3(10^2^,10^3^,10^3^)** | **3(10^2^,10^2^,10^2^)** | **3(10^2.5^,10^2.5^,10^2.33^)**^c^ | **3(10^2.33^,10^2.5^,10^2.5^)** | **1(10^3.33^)** | **3(10^3.33^,10^3^,10^3.5^)** |
|  |  | **3(2.55×10^3^,1.26×10^3^, 8.30×10^2^)** | **3(1.20×10^2^,1.64×10^3^, 8.60×10^2^)** | **3(4.85×10^3^,3.90×10^2^, 4.70×10^2^)** | **3(8.80×10^3^,5.94×10^3^,2.13×10^4^)**^d^ | **3(8.80×10^2^,1.08×10^3^,1.51×10^3^)** | **1(5.20×10^4^)** | **3(1.15×10^4^,9.15×10^3^,1.55×10^4^)** |
| **3** | **TCID_50_**  **qPCR** | **3(10^2.5^,10^2.5^,10^2.5^)** | **3(10^3.5^,10^3.5^,10^3.5^)** | **3(10^2^,10^2.5^,10^2.5^)** | **3(10^3.67^,10^4.67^,10^4.67^)** | **3(10^2.5^,10^2.5^,10^2.5^)** | **1(10^2.5^)** | **3(10^3.33^,10^3.33^,10^3^)** |
|  |  | **3(8.20×10^2^,2.02×10^3^,8.90×10^3^)** | **3(2.69×10^3^,1.036×10^4^,8.19×10^3^)** | **3(7.95×10^3^,6.29×10^3^,7.43×10^3^)** | **3(1.45×10^5^,8.12×10^4^,7.79×10^4^)** | **3(2.36×10^3^,2.33×10^3^,2.28×10^3^)** | **1(2.64×10^4^)** | **3(1.41×10^4^,3.75×10^3^,3.55×10^3^)** |
| **5** | **TCID_50_**  **qPCR** | **3(10^3^,10^2.5^,10^3.33^)** | **3(10^4.5^,10^4.33^,10^4.5^)** | **3(10^2.5^,10^2.67^,10^2.67^)** | **3(10^3^,10^4.5^,10^4.5^)** | **3(10^3.33^,10^3.5^10^3.5^)** | **1(10^3^)** | **3(10^3.33^,10^3^ 10^3^)** |
|  |  | **3(4.93×10^4^,5.66×10^5^,7.46×10^4^)** | **3(2.96×10^3^,4.06×10^4^,2.66×10^4^)** | **3(3.11×10^4^,7.26×10^4^,7.60×10^4^)** | **3(5.73×10^4^,2.77×10^4^,1.01×10^4^)** | **3(5.19×10^4^,1.69×10^5^,1.60×10^5^)** | **1(3.55×10^4^)** | **3(2.98×10^4^,1.18×10^4^,7.29×10^3^)** |
| **7** | **TCID_50_**  **qPCR** | **3(10^2.67^,10^2.67^,10^2.5^)** | **3(10^3.5^,10^3^,10^3^)** | **3(10^2.33^,10^2.33^,10^2.5^)** | **3(10^2.5^,10^2.33^,10^2.33^)** | **3(10^3.33^,10^3.33^,10^3.5^)** | **1(10^2.33^)** | **3(10^3^,10^3.33^,10^3.33^)** |
|  |  | **3(1.12×10^3^,1.09×10^3^,7.08×10^3^)** | **3(1.45×10^3^,1.27×10^3^,6.36×10^3^)** | **3(1.07×10^3^,7.21×10^3^,3.37×10^3^)** | **3(5.38×10^3^, 6.74×10^3^, 8.27×10^3^)** | **3(6.90×10^3^,6.51×10^3^,5.36×10^3^)** | **1(5.65×10^3^)** | **3(1.18×10^3^,1.98×10^4^,1.55×10^4^)** |
| **9** | **TCID_50_**  **qPCR** | **3(10^2.33^,10^2.33^,10^2.5^)** | **3(10^3^,10^3^,10^3^)** | **3(10^2.67^,10^2.5^,10^2.5^)** | **3(10^2.5^,10^2.5^,10^2.5^)** | **3(10^3.33^,10^3.5^,10^3.5^)** | **1(10^2.66^)** | **3(10^3.33^,10^3.5^,10^3.33^)** |
|  |  | **3(1.04×10^3^,7.52×10^3^,1.04×10^3^)** | **3(1.16×10^3^,1.24×10^3^,1.34×10^3^)** | **3(6.61×10^3^,1.32×10^4^,6.29×10^3^)** | **3(9.63×10^3^,4.03×10^3^, 8.18×10^3^)** | **3(7.60×10^2^,3.79×10^3^,3.04×10^3^)** | **1(3.62×10^4^)** | **3(2.44×10^4^,7.85×10^3^,1.61×10^4^)** |
| **11** | **TCID_50_**  **qPCR** | **3(10^2.5^,10^2.5^,10^2.5^)** | **3(10^3.5^,10^3.5^,10^3.33^)** | **3(10^2.5^,10^2.5^,10^2.67^)** | **3(10^2^,10^2.5^,10^2.5^)** | **3(10^3.5^,10^3.5^,10^3.5^)** | **1(10^3.33^)** | **3(10^3.33^,10^3^,10^3.33^)** |
|  |  | **3(1.64×10^3^,3.43×10^3^,1.83×10^3^)** | **3(2.24×10^3^,2.06×10^3^,1.39×10^3^)** | **3(4.04×10^3^, 1.69×10^3^,4.32×10^3^)** | **3(2.23×10^3^, 3.88×10^3^, 3.70×10^3^)** | **3(3.14×10^3^,1.93×10^3^,1.29×10^3^)** | **1(5.75×10^4^)** | **3(1.28×10^4^,1.32×10^4^,2.38×10^4^)** |
| **13** | **TCID_50_**  **qPCR** | **3(10^2^,10^2^,10^1.33^)** | **3(10^2.67^,10^3^,10^3.33^)** | **3(10^2.5^,10^2.5^,10^2.5^)** | **3(10^2.67^,10^2.5^,10^2.5^)** | **3(10^3^,10^3^,10^3^)** | **1(10^4^)** | **3(10^3.67^,10^3.5^,10^3.5^)** |
|  |  | **3(3.29×10^3^,1.10×10^3^,1.66×10^3^)** | **3(1.22×10^3^,9.80×10^2^,7.30×10^2^)** | **3(6.51×10^3^,4.84×10^3^,1.11×10^3^)** | **3(1.16×10^4^,4.99×10^3^,4.51×10^3^)** | **3(2.64×10^3^,2.47×10^3^,1.27×10^3^)** | **1(9.82×10^4^)** | **3(4.26×10^4^,3.10×10^4^,2.27×10^4^)** |
| **15** | **TCID_50_**  **qPCR** | **3(10,10,10)** | **3(10^2^,10^2^,10^2.5^)** | **3(10^1.33^,10,10)** | **3(10^2.67^,10^2.5^,10^3^)** | **3(10^2.5^,10^2.33^,10^2.33^)** | **1(10^4.33^)** | **3(10^3.5^,10^3.33^,10^3^)** |
|  |  | **3(1.46×10^3^,1.33×10^3^,4.03×10^3^)** | **3(1.25×10^3^,7.60×10^2^,8.80×10^2^)** | **3(6.60×10^2^,1.90×10^2^,3.60×10^2^)** | **3(4.56×10^3^,6.63×10^3^,1.21×10^4^)** | **3(3.41×10^3^,1.73×10^3^,1.73×10^3^)** | **1(1.17×10^5^)** | **3(2.94×10^4^,1.84×10^4^,2.92×10^4^)** |

**Table S1.** **Results of viral titers (TCID_50_) and viral loads of tissue homogenates of mice^a^**

^a^ Three animals were tested for two tests. Iso: virus isolation; qPCR: Real-time PCR. ^b^ Days post inoculation when euthanized.

^c^ Positive number and TCID_50_ of tissue homogenates per ml. ^d^ Positive number and viral load of tissue homogenates per gram.
